# Normative data for interorbital distance in a paediatric Caucasian population

**DOI:** 10.1007/s00276-026-03849-3

**Published:** 2026-02-27

**Authors:** Mathilde Pech de Laclause, Baptiste Morel, Boris Laure, Anne Morice

**Affiliations:** 1 Department of Paediatric Maxillofacial Surgery and Craniofacial Surgery, Reference Center for Rare Craniofacial Malformations, Clocheville Hospital, Tours University Hospital, Tours, France; 2 Department of Paediatric Radiology, Clocheville Hospital, Tours University Hospital, Tours, France; 3 Maxillo-facial and cranio-facial surgery unit, Hôpital Clocheville, CHRU de Tours, 47 boulevard Béranger, Tours, 37000 France

**Keywords:** Interorbital distance, CT-scans, orbital growth, paediatric population, Caucasian

## Abstract

**Background:**

Orbital growth represents a key component of craniofacial development to investigate due to the many craniofacial abnormalities which can interfere with it. Defining orbital growth patterns is essential for distinguishing physiological from pathological orbital development. Normative orbital growth data in Caucasian paediatric populations remain scarce.

**Objective:**

Our study aimed to determine interorbital distance in a healthy Caucasian children population.

**Materials and methods:**

A retrospective study was conducted in France, based on craniofacial CT scans measurements in patients aged from 3 months to 10 years. Measurements were taken at the neuro-ocular plane and included the bony inner interorbital distance (IOD), bony lateral orbital distance (LOD), and IOD/LOD ratio. Mean +/- SD was calculated for each age group, and comparisons between sexes and age groups were performed using the Mann–Whitney test (*p* < 0.05).

**Results:**

A total of 466 CT-scans were analysed (216 females and 250 males). The mean IOD increased progressively, from 18.76 +/- 1.17 mm at 3 months to 22.79 +/- 1.73 mm at 10 years of age. The most important increase of IOD and LOD values was observed during the first 2 years of age (*p* < 0.01) and between 7 and 8 years of age (*p* < 0.05). The IOD/LOD ratio remained constant with age, showing isometric growth of the orbits.

**Conclusion:**

This study provides detailed normative data of orbital measurements in Caucasian children and highlights two critical periods of accelerated orbital growth.

**Supplementary Information:**

The online version contains supplementary material available at 10.1007/s00276-026-03849-3.

## Background

The pattern of human orbital growth is an important point of facial development to investigate, due to the many craniofacial abnormalities requiring surgical procedures which can interfere with it. Interorbital distance can be abnormally increased or decreased in case of hypertelorism or hypotelorism, respectively.

Orbital hypotelorism is characterized by reduced interorbital distances, it is often associated with syndromic anomalies [17]. Trigonocephaly is one of the most frequent craniofacial malformations leading to hypotelorism [10]. It results from the premature closure of the metopic suture causing the inability of the frontal bones to grow laterally, and subsequent reduced interorbital distance [12]. Hypotelorism is also described in more than 60 different associated conditions, such as holoprosencephaly, trisomy 13, Coffin-Siris syndrome, Meckel-Gruber syndrome, or Williams syndrome [2, 13, 16, 19].

Orbital hypertelorism, firstly described by Greig in 1924, corresponds to the true lateralization of the entire orbital complex, characterized by excessive interorbital inner and lateral orbital distances [11]. Hypertelorism is observed in various congenital and tumoral craniofacial diseases, such as craniofrontonasal dysplasia, median and paramedian craniofacial clefts, syndromic craniosynostosis, frontonasoethmoidal tumors, or encephaloceles [26, 28].

A good knowledge of physiological orbital growth is essential to better assess normal and abnormal orbital development and will help clinicians and craniofacial surgeons to guide clinical evaluation and surgical planning in children with craniofacial abnormalities affecting the orbits. In fact, surgical correction of craniofacial anomalies interfere with orbital growth, and age at surgery for the correction of hypertelorism is controversial [21, 22]. Applying an overcorrection of the interorbital distance based on normal values according to the age at surgery, may prevent the risk of relapse particularly in case of early surgical correction [22].

Several studies report normative values of interorbital distance based on radiographic 2D measurements [15]. However, CT-scan measurements allow more reliable and reproducible measurements than cephalometric measures.

Beyond its clinical relevance, knowledge of orbital development also holds anthropological and forensic values. Establishing reliable, population-specific reference values for orbital growth is necessary, as craniofacial norms differ across ethnic groups [1, 15, 29]. Several studies report Asian and American facial and orbital growth characteristics in healthy populations, but to date, there is a lack of data reporting normal orbital growth patterns in Caucasian populations [3, 4, 8, 14, 18, 20, 23, 24, 30].

The aim of our study was to analyse orbital bone growth based on CT-scan measurements in a healthy paediatric Caucasian population.

## Materials and methods

Selection of the craniofacial CT-scans used for the analyses was based on a list provided by the Department of Paediatric Radiology (BM) of our center; this list reviewed all craniofacial CT-scans retrospectively performed in France, between 2010 and 2024, in patients aged from 3 months to 10 years.

All the CT-scans were produced by using the same multidetector CT-scan machine (SIEMENS ^®^ SOMATOM DEFINITION EDGE, 2020) with high-resolution contiguous sections in an axial plane (slice thickness 0.6 mm). CT images were analyzed in bone window, and the image acquisition protocol was identical throughout the study period. We excluded all patients with a known facial fracture, craniofacial syndrome, infectious or tumoral diseases or previous facial surgery which affect the orbito-facial studied area. CT scans exhibiting motion artifacts were excluded from the analysis.

**Fig. 1:**
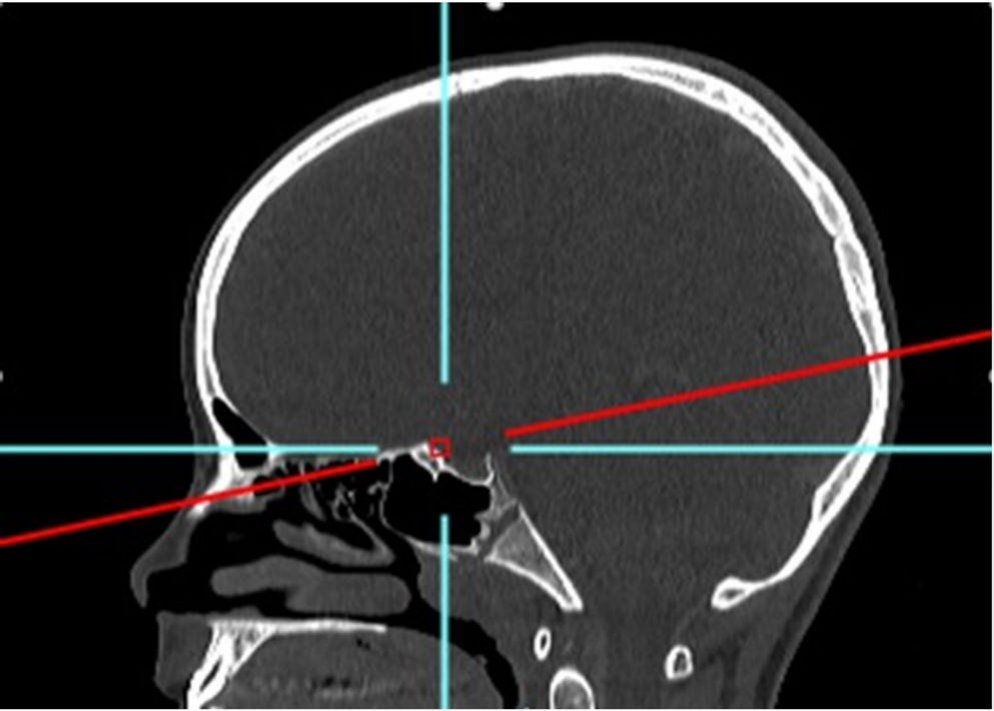
Craniofacial CT-scan, sagittal view. The red line represents the level of neuro-ocular plane as described by Cabanis et al. [25, 26]

**Fig. 2:**
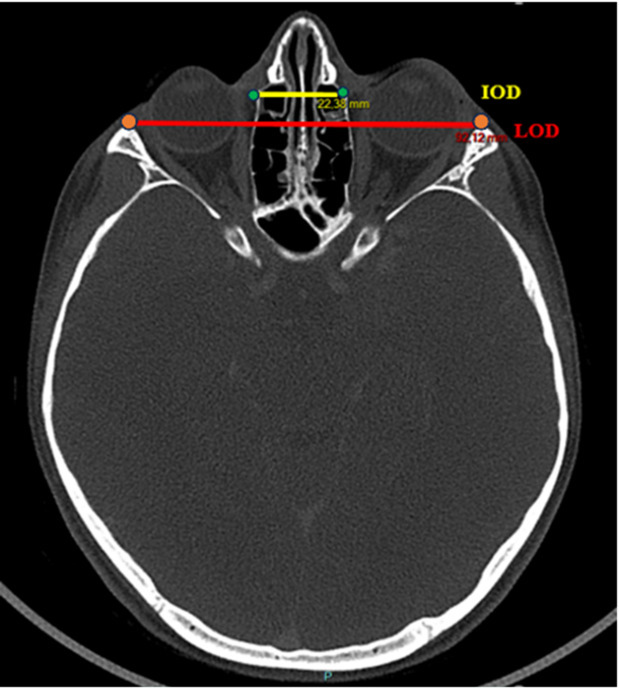
Craniofacial CT-scan, axial view (Cabanis plane). Bony inner interorbital (IOD) (yellow line) and bony lateral orbital (LOD) (red line) distances [25, 26]. Green point = dacryon; orange point = most anterior point at the level of the fronto-zygomatic suture

Age groups were categorized as follows: 3–6 months, 6–12 months, 12–18 months, 18–24 months, and then every year until age 10–11 years of life. The anatomical landmarks used to evaluate interorbital distance were determined by the craniofacial surgeons of our team (BL, AM), in collaboration with our referring paediatric radiologist (BM) as follows: bony inner interorbital distance (IOD), bony lateral orbital distance (LOD) and the IOD/LOD ratio. These anatomical landmarks were placed at the level of the neuro-ocular plane as described by Cabanis et al., which is routinely used and taught by clinicians in our department [5, 6]. This plane is defined by the center of the pupil and the intracranial optic axis, reflecting the functional orientation of the visual system. The IOD was defined as the distance between the two dacryons and the LOD was defined as the distance between the lateral margins of the frontozygomatic sutures. All measurements were performed by the lead author (MPL) to minimize rater error in data collection. The measurements were carried out at least two times and the average was calculated. A third reading was performed when intra-observer variability exceeded 5% between the two measurements. All image manipulations and measurements were made using the PACS Carestream Philips (Figs. 1 and 2).

### Statistical analysis

Mean +/- SD was calculated for inner IOD, LOD, and IOD/LOD ratio. Male/Female ratio was evaluated for each age group. Comparisons of inner IOD, LOD and IOD/LOD ratio between each age group and between males and females were conducted (Mann Whitney test). The Mann–Whitney test was chosen because our data were not normally distributed. Intraclass correlation coefficients were calculated for IOD and LOD for each age group. Statistical analyses were performed using Excel^®^ and Python^®^. The threshold for statistical significance was set to *p* < 0.05: *0.01 < *p* < 0.05; **0.001 < *p* < 0.01; ***p < 0.0001.

## Results

In total, 466 Caucasian subjects were included in this study, including 216 (46%) females and 250 males (54%). Age at evaluation ranged from 3 months to 10.5 years. The average size of groups was 36 subjects (+/- 4), ranging from 21 to 45 subjects per group, without any significant differences between male and female rates (*p* = 0.066) for each age group. The two most common indications for CT-scan acquisition were the evaluation of unexplained headaches and head trauma. No underlying orbital or craniofacial pathology that could affect orbital measurements was identified. Intravenous contrast administration was used in some examinations according to clinical indication; however, contrast enhancement does not influence bone-window measurements of the interorbital distances. Repeated CT-scan examinations at different ages were performed in 38/466 patients (7.2%) because of their underlying pathology; however, 37 of these patients underwent only two CT-scans at different ages. These cases were not representative of the overall study population.

Intraclass correlation coefficients for IOD and LOD measurements ranged between 0.95 and 0.99 for each age groups (Supplemental Table).

**Fig. 3:**
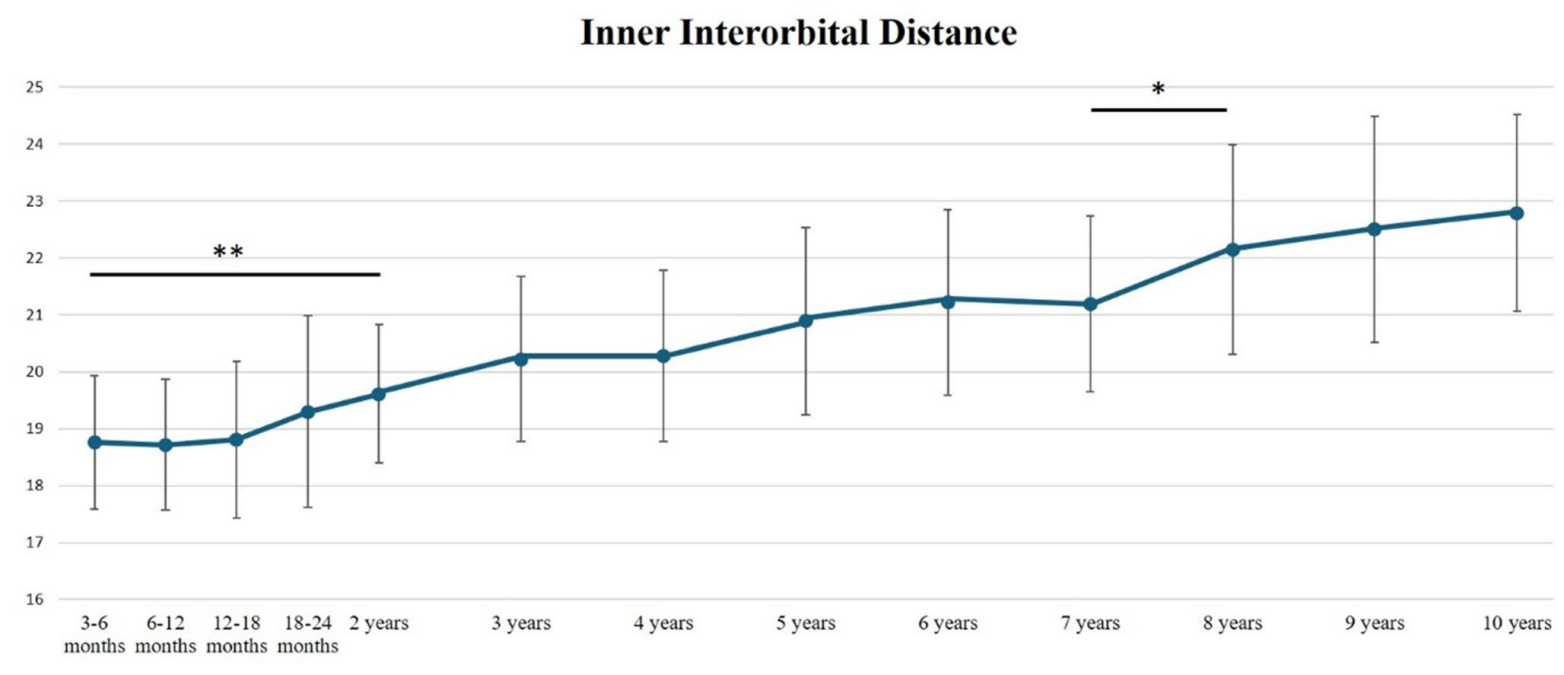
Inner Interorbital Distance (IOD) from 3 months to 10 years of age. Mean +/- SD (mm). (*0.01 < *p* < 0.05; **0.001 < *p* < 0.01)

The mean inner interorbital distance (IOD) increased progressively with age, from 18.76 +/- 1.17 mm at 3 months of age to 22.79 +/- 1.73 mm at 10 years of age, with an average increase of 2% between age groups (from 0.3 to 4.53%) (Fig. 3). The highest increase of inner IOD values was observed during the first 2 years of age and between 7 and 8 years of age, with statistically significant differences between these age groups: between the age of 3–6 months and the age of 2 years, the mean increase of IOD distance was 4.53%, *p* = 0.004; and between the age of 7 years and the age of 8 years, the mean increase of IOD distance was + 4.53%, *p* = 0.042 (Table 1; Fig. 3).

**Table 1:** Bony inner interorbital distances (IOD) values by age group and sex, and comparisons between males and females (*)

Age group	Number of Subjects per group	Inner Interorbital distance (IOD) Mean +/- Standard Deviation (mm)			
		All (mean +/- SD)	F (mean +/- SD)	M (mean +/- SD)	*p*-Value*
3–6 mo	33 (13 F, 20 M)	18.76 +/- 1.17	18.58 +/- 1.25	18.89 +/- 1.13	0.519
6–12 mo	45 (19 F, 26 M)	18.72 +/- 1.15	18.37 +/- 0.96	18.94 +/- 1.20	0.105
12–18 mo	38 (17 F, 21 M)	18.81 +/- 1.38	18.44 +/- 1.27	19.11 +/- 1.41	0.161
18–24 mo	21 (11 F, 10 M)	19.30 +/- 1.68	19.27 +/- 1.86	19.34 +/- 1.55	0.918
2–3 y	39 (16 F, 23 M)	19.61 +/- 1.21	19.48 +/- 1.29	19.71 +/- 1.18	0.525
3–4 y	40 (17 F, 23 M)	20.22 +/- 1.45	19.76 +/- 1.28	20.57 +/- 1.49	0.120
4–5 y	35 (17 F, 18 M)	20.28 +/- 1.50	20.11 +/- 1.38	20.45 +/- 1.62	0.586
5–6 y	35 (17 F, 18 M)	20.89 +/- 1.65	20.31 +/- 1.43	21.45 +/- 1.68	0.041*
6–7 y	37 (20 F, 17 M)	21.22 +/- 1.63	21.11 +/- 1.61	21.34 +/- 1.69	0.715
7–8 y	36 (20 F, 16 M)	21.19 +/- 1.54	21.70 +/- 1.58	20.55 +/- 1.26	0.030*
8–9 y	33 (15 F, 18 M)	22.15 +/- 1.84	21.94 +/- 1.86	22.33 +/- 1.86	0.613
9–10 y	42 (18 F, 24 M)	22.50 +/- 1.99	22.06 +/- 1.77	22.84 +/- 2.11	0.438
10–11 y	32 (16 F, 16 M)	22.79 +/- 1.73	22.03 +/- 1.43	23.55 +/- 1.71	0.009**

F = females, M = males, y = years, mo = months

Statistical differences between male and females: *0.010 < *p* < 0.050; **0.001 < *p* < 0.010; ***p < 0.0001

**Fig. 4:**
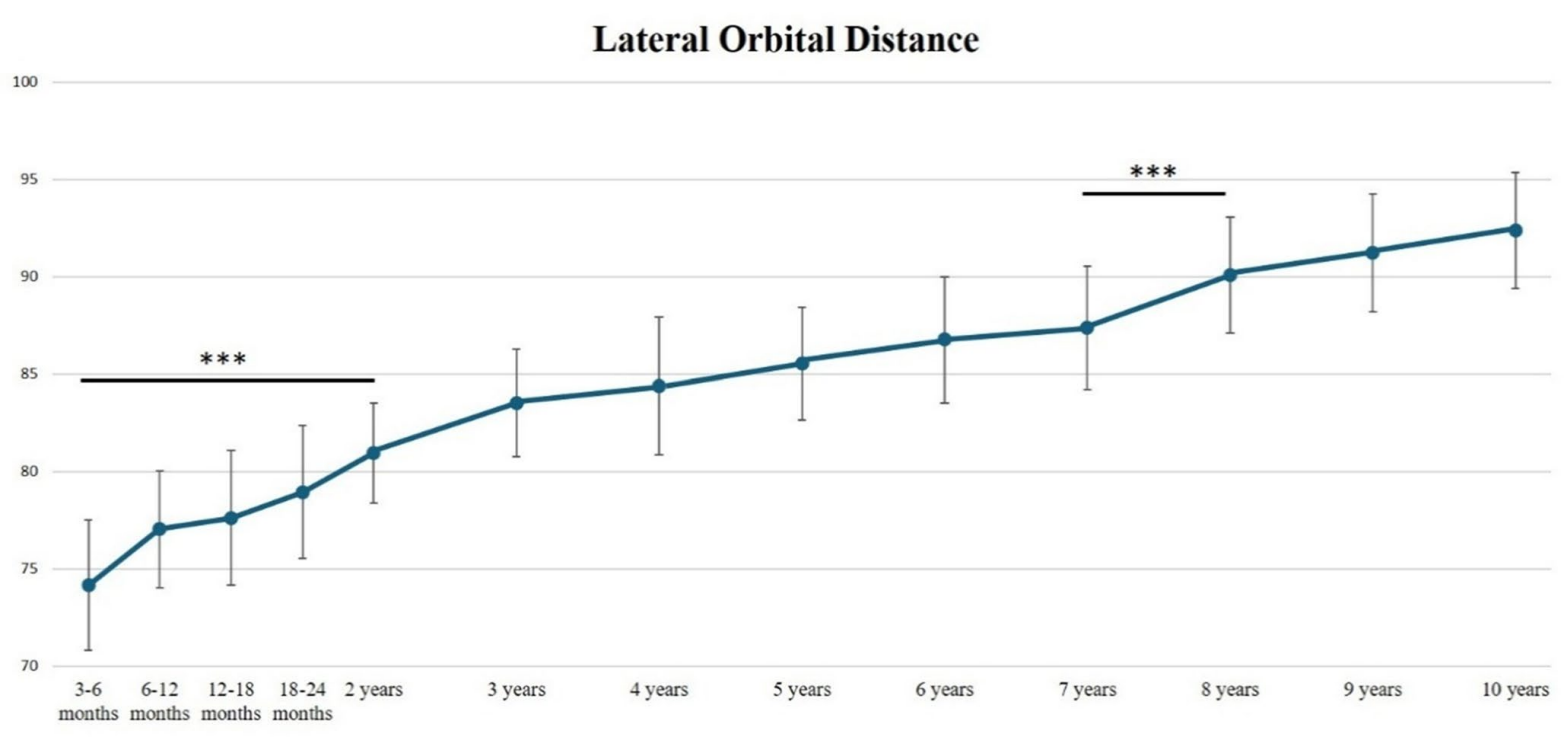
Lateral Orbital Distance (LOD) from 3 months to 10 years of age. Mean +/- SD (mm). (***p < 0.0001)

Concerning the lateral orbital distance (LOD), the highest increase was observed during the first 2 years of age (+ 9.11%, *p* < 0.001), and between the age of 7 years and the age of 8 years (+ 3.1%, *p* < 0.001) (Table 2; Fig. 4).

**Table 2:** Lateral orbital distance (LOD) values by age group and sex, and comparisons between males and females (*)

Age group	Number of CT-scans per group	Lateral Orbital distance (LOD) Mean +/- Standard Deviation (mm)			
		All (mean +/- SD)	F (mean +/- SD)	M (mean +/- SD)	*p*-Value
3–6 mo	33 (13 F, 20 M)	74.17 +/- 3.35	72.02 +/- 2.62	75.65 +/- 3.06	0.002**
6–12 mo	45 (19 F, 26 M)	77.04 +/- 3.00	76.03 +/- 2.71	77.77 +/- 3.03	0.030*
12–18 mo	38 (17 F, 21 M)	77.62 +/- 3.48	76.68 +/- 3.57	78.38 +/- 3.29	0.123
18–24 mo	21 (11 F, 10 M)	78.94 +/- 3.42	77.93 +/- 3.86	80.05 +/- 2.60	0.132
2–3 y	39 (16 F, 23 M)	80.93 +/- 2.57	80.39 +/- 1.96	81.31 +/- 2.90	0.404
3–4 y	40 (17 F, 23 M)	83.52 +/- 2.77	81.90 +/- 1.76	84.71 +/- 2.80	0.001**
4–5 y	35 (17 F, 18 M)	84.38 +/- 3.54	82.97 +/- 3.41	85.70 +/- 3.21	0.052
5–6 y	35 (17 F, 18 M)	85.53 +/- 2.90	84.39 +/- 2.74	86.60 +/- 2.69	0.044
6–7 y	37 (20 F, 17 M)	86.76 +/- 3.25	86.03 +/- 2.67	87.62 +/- 3.72	0.198
7–8 y	36 (20 F, 16 M)	87.36 +/- 3.17	87.38 +/- 2.91	87.33 +/- 3.57	0.836
8–9 y	33 (15 F, 18 M)	90.07 +/- 2.97	89.40 +/- 3.64	90.62 +/- 2.22	0.556
9–10 y	42 (18 F, 24 M)	91.23 +/- 3.01	90.29 +/- 2.62	91.93 +/- 3.14	0.220
10–11 y	32 (16 F, 16 M)	92.37 +/- 2.98	91.06 +/- 2.45	93.68 +/- 2.96	0.023*

F = females, M = males, y = years, mo = months

Statistical differences between male and females: *0.010 < *p* < 0.050; **0.001 < *p* < 0.010; ***p < 0.0001

The mean IOD/LOD ratio was 0.24 +/- 0.004 mm; it remained constant with age, without significant differences between each age groups (Table 3).

**Table 3:** Bony inner interorbital distance (IOD) to lateral orbital distance values (LOD) ratio by age group and sex and comparisons between males and females (*)

Age group	Number of CT-scans per group	Inner Interorbital / Lateral Orbital distances ratio (IOD/LOD ratio)			
		All (mean +/- SD)	F (mean +/- SD)	M (mean +/- SD)	*p*-Value*
3–6 mo	33 (13 F, 20 M)	0.25 +/- 0,01	0.26	0.25	0.610
6–12 mo	45 (19 F, 26 M)	0.24 +/- 0,01	0.24	0.24	0.912
12–18 mo	38 (17 F, 21 M)	0.24 +/- 0,01	0.24	0.24	0.881
18–24 mo	21 (11 F, 10 M)	0.24 +/- 0,02	0.25	0.24	0.772
2–3 y	39 (16 F, 23 M)	0.24 +/- 0,01	0.24	0.24	0.952
3–4 y	40 (17 F, 23 M)	0.24 +/- 0,01	0.24	0.24	0.944
4–5 y	35 (17 F, 18 M)	0.24 +/- 0,01	0.24	0.24	0.826
5–6 y	35 (17 F, 18 M)	0.24 +/- 0,02	0.24	0.25	0.764
6–7 y	37 (20 F, 17 M)	0.24 +/- 0,02	0.25	0.24	0.834
7–8 y	36 (20 F, 16 M)	0.24 +/- 0,01	0.25	0.24	0.582
8–9 y	33 (15 F, 18 M)	0.25 +/- 0,02	0.25	0.25	0.976
9–10 y	42 (18 F, 24 M)	0.25 +/- 0,02	0.24	0.25	0.653
10–11 y	32 (16 F, 16 M)	0.25 +/- 0,02	0.24	0.25	0.617

F = females, M = males, y = years, mo = months

*0.010 < *p* < 0.050; **0.001 < *p* < 0.010; ***p < 0.0001

Considering IOD, a significant difference between females and males was found at 5–6, 7–8 and 10–11 years of age. In these age groups, males had a wider IOD distance (+ 1 to 2 mm when compared with female values, *p* = 0.041, *p* = 0.03, *p* = 0.009, respectively) (Table 1). Considering lateral orbital distance, a significant difference between females and males was found at 3–6 months, 6–12 months, 3 years and 10 years of age. In these age groups, males had a wider LOD (+ 1 to 3 mm when compared with female values, *p* = 0.002, *p* = 0.03, *p* = 0.001, *p* = 0.023, respectively) (Table 2). No statistically significant difference between females and males was observed concerning IOD/LOD ratio for each age group (Table 3).

**Fig. 5:**
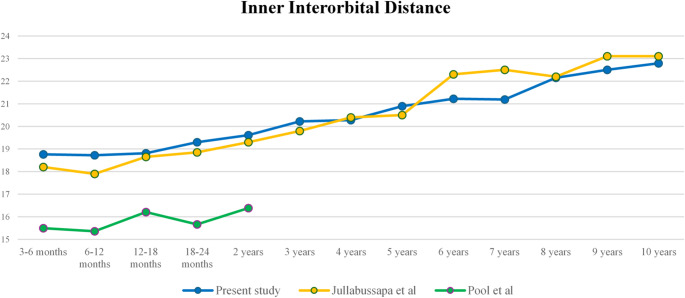
Comparisons of Inner Interorbital distance values between our series and previous published series. Mean in mm [17, 18]

## Discussion

Craniofacial morphology and growth have traditionally been studied using radiographic 2D measurements, and adult population has been more widely described than paediatric ones [15]. CT-scan is now available in routine and provides more reliable and reproducible 3D measurements than 2D radiographic-based measurements.

Asian and American facial and orbital growth characteristics are well reported in literature [3, 4, 8, 14, 18, 20, 23, 24, 30]. However, craniofacial morphology varies across ethnicities, and, to date, there is still a lack of data concerning normal orbital growth patterns in Caucasian populations [18, 24].

Based on 3D CT-scan measurements in a paediatric Caucasian population in France, we showed that inner interorbital distances increased progressively during growth, from 3 months to 10 years of age, with an average increase of 2% +/- 1.3 per year. Two growth spurts were observed considering both inner and lateral orbital distances, during the first 2 years of life, and between 7 and 8 years of age, highlighting accelerated orbital development at these ages. A similar growth pattern was also reported by Chatdokmaiprai et al. [8].

According to Tessier et al., mean inner interorbital distance reaches 25 mm in females and 28 mm males at adult age [28]. When compared with these adults’ values, mean inner interorbital distance reached approximately 78% and 89% of the adult values, at the age of 2 years, and 8 years, respectively, in our series. Then, the orbital growth slowed down since it reached at the age of 10 years, almost 91% of the adult values, as reported previously [3, 14, 18]. Ye et al. reported also 2 growth spurts during orbital growth: during the first 2 years and between the age of 5 and 13 years [30]. In addition, our study showed constant IOD/LOD values, demonstrating isometric orbital and zygomatic growth.

Several factors influence orbital development and growth and determine final orbital size, volume and shape. Embryology and fetal development of the orbits begins at the time of the neurulation during week 4 of embryogenesis [3]. The development of the eyeballs establishes the scaffolding of the orbital bones. The absence of a normally sized globe may lead to underdevelopment of the orbital cavity and surrounding soft tissues [9]. Convergence of the orbits pre- and postnatally determines the final position of the orbits and interorbital distances: at 2 months of gestation, orbits have an initial 180 degrees relation to each other, whereas they reach a 71 degrees relation to each other at birth. Orbital development is also linked to the development of the brain and of the anterior cranial vault and skull base [7, 27]. Cranial growth rate is most significant during the first two years of life. By the age of two, the head circumference is estimated to be very close to adult size, reaching 87% of the adult values [25]. This is consistent with our findings concerning interorbital distance, as we demonstrated that it reaches nearly 80% of the adult values at 2–3 years of age. In addition, orbital growth and shape is influenced by sinus pneumatisation in the postnatal period [3]. Orbital growth and interorbital distance also depends on the patency of cranial sutures [7, 27]. The time of cranial suture closure depends on the suture site and highly varies among healthy populations. Noticeably, premature fusion of coronal sutures, causing plagiocephaly and brachycephaly, leads to reduced orbital volume and is often associated with hypertelorism, whereas, in case of premature fusion of the metopic suture, leading to trigonocephaly, triangular frontal shape and reduced interorbital distance are observed [10, 25].

We found some discrepancies between males and females concerning inner and lateral interorbital distances, this was also reported in other studies, but at different ages [4, 24]. Noticeably, some studies did not observe any significant differences between males and females, either in pediatric and/or adult populations [4, 8, 14]. The differences observed between the series may be due to the inclusion bias due to the CT-scan selection and the sample size. However, when a significant difference was observed, wider interorbital distances most often involved males [4, 24]. Variations concerning orbital dimensions between males and females and between Caucasian and Black populations in adults have been reported in anthropological literature. Orbital height was found to be greater in males compared to females, particularly among White South Africans [1, 29].

When comparing our results with those reported from the study of Asian populations (Thailand), we found a similar orbital growth pattern with a rapid increase of interorbital distance values during the first 2 years of life [18]. When compared with a study conducted in American population (USA), we showed slightly higher values for inner and lateral interorbital distances, but growth curves and inner/lateral orbital distance ratio remained similar (Fig. 5) [24].

In this present study, interorbital distances measurements were performed at the level of neuro-ocular plane as described by Cabanis et al. [5, 6]. This neuro-ocular plane is a standard for the CT-scan or MRI study of the visual pathway; it is the permanent orientation of the brain in space and replicates the Broca’s visual plan. In some other studies, the Frankfort horizontal plane was used for interorbital distance measurements [18, 24, 30]. This plane is defined by the highest point on the upper margin of the opening of each external auditory canal and the lowest point on the lower margin of the left orbit. Despite the different planes used within the different series and ours, interorbital distance showed similar evolution during growth and inner/lateral interorbital distance ratio remained constant with age and comparable. We chose the Cabanis’ plane, rather than Frankfort ones, as we obtained better reproducibility of interorbital distance measurements, with a low intra-observer variability and high intraclass correlation coefficients.

Our study has some limitations that must be considered. Firstly, our samples came from a single tertiary care center and this population may differ from the general healthy population. Secondly, although the average size of our groups is comparable to those of other studies, the sample size may limit statistical power of this study. Thirdly, due to the retrospective design of our study, we could not provide longitudinal data for orbital measurements from the same patients during growth. In future, longitudinal prospective studies could help to better identify factors that influence orbital growth among healthy individuals.

Overall, our study provides additional normative values for interorbital distance in a Caucasian paediatric population, that are valuable not only for clinicians but also for forensic pathologists and anthropologists. From a clinical point of view, our findings suggest that surgical planning for craniofacial anomalies affecting the orbits should consider age-specific normative data as well as ethnical origins, as normative values may vary across populations.

## Conclusion

These normative data concerning orbital bone growth in Caucasian paediatric population, will help in future to better define the interorbital distance to apply per-operatively for the correction of hypertelorism, according to the patient’s age at the time of surgery. Beyond their clinical relevance, these findings also provide valuable anthropological reference data for the assessment of normal and pathological craniofacial development in growing individuals.

## Supplementary Information

Below is the link to the electronic supplementary material.

Supplementary Material 1

## Data Availability

No datasets were generated or analysed during the current study.

## Abbreviations

IOD: Inner Interorbital Distance
LOD: Lateral Orbital Distance
ICC: Intraclass Correlation Coefficient
