# Supplementary material for: Normative data for interorbital distance in a paediatric Caucasian population
Source: Surg Radiol Anat. 2026 Feb 27;48(1):76. doi: 10.1007/s00276-026-03849-3 (PMC12948904; doi:10.1007/s00276-026-03849-3)
Supplement: Supplementary file 1 — Supplementary Material 1 [file 276_2026_3849_MOESM1_ESM.docx]

Supplemental Table: Intraclass Correlation Coefficients for Inner Interorbital Distance measurements.

| Age | **Intraclass Correlation Coefficient (ICC)** | | |
| --- | --- | --- | --- |
|  | All | Females | Males |
| 3-6 mo | 0.98 | 0.99 | 0.98 |
| 6-12 mo | 0.96 | 0.94 | 0.97 |
| 12-18 mo | 0.97 | 0.96 | 0.97 |
| 18-24 mo | 0.98 | 0.98 | 0.98 |
| 2 y | 0.96 | 0.97 | 0.95 |
| 3 y | 0.97 | 0.96 | 0.97 |
| 4 y | 0.95 | 0.94 | 0.96 |
| 5 y | 0.97 | 0.96 | 0.96 |
| 6 y | 0.97 | 0.98 | 0.96 |
| 7 y | 0.98 | 0.99 | 0.95 |
| 8 y | 0.98 | 0.97 | 0.98 |
| 9 y | 0.99 | 0.99 | 0.98 |
| 10 y | 0.98 | 0.98 | 0.98 |
